# A Rare Case of Co-existent Superior Semicircular Dehiscence and Sigmoid Sinus Plate Dehiscence Contributing to Pulsatile Tinnitus

**DOI:** 10.7759/cureus.84477

**Published:** 2025-05-20

**Authors:** Mounika Reddy Y., Anoop Kumar Pandey

**Affiliations:** 1 Otolaryngology - Head and Neck Surgery, Zulekha Hospital, Dubai, ARE; 2 Radiology, Zulekha Hospital, Dubai, ARE

**Keywords:** deafness, pulsatile tinnitus, sigmoid sinus plate dehiscence, superior semicircular canal dehiscence, temporal bone imaging

## Abstract

Pulsatile tinnitus (PT) is an uncommon but often debilitating symptom, frequently associated with vascular etiologies, tumors, or structural abnormalities of the temporal bone. We present the case of a 40-year-old woman diagnosed with concurrent unilateral superior semicircular canal dehiscence (SSCD) and sigmoid sinus plate dehiscence (SSPD), both contributing to her symptoms. This case highlights the critical role of thorough clinical and radiological assessment in detecting multiple coexisting etiologies in patients with PT.

## Introduction

Tinnitus is defined as the perception of sound originating internally, in the absence of any external auditory stimulus. Pulsatile tinnitus (PT) is a distinct subtype, characterized by a rhythmic sound that is synchronous with the patient's heartbeat [1]. PT can significantly affect a patient’s mental health, contributing to stress, depression, and anxiety. It may result from a variety of underlying pathological conditions, including vascular anomalies such as arteriovenous fistulas/malformations, jugular vein compression, emissary veins, and venous sinus stenosis or diverticula; tumors such as skull base schwannomas, meningiomas, paragangliomas, and hemangiomas; and bony abnormalities like otosclerosis, superior semicircular canal dehiscence (SSCD), and sigmoid sinus plate dehiscence (SSPD) [2].

A comprehensive clinical and radiological evaluation is essential to identify the underlying cause of PT. Auscultation should be performed to detect any associated bruits, and a thorough otological examination is necessary to assess for concurrent hearing loss. Radiological imaging plays a pivotal role, as a specific causative abnormality can be identified in more than 75% of PT cases. High-resolution computed tomography (CT) of the temporal bone is the modality of choice for evaluating bony abnormalities, whereas magnetic resonance imaging (MRI) combined with MR angiography or venography is preferred for assessing vascular and neoplastic causes [3]. Conventional digital subtraction angiography may also be employed for more detailed evaluation of vascular anomalies.

## Case presentation

A 40-year-old female presented with a six-month history of right-sided pulsatile tinnitus, described as a rhythmic sound synchronous with her heartbeat. She also reported progressive hearing loss in the right ear and mild aural fullness on the same side. She denied experiencing vertigo or otalgia. There was no significant medical history, history of ear trauma, prior otologic surgeries, or any history of previous ototoxic medication.

**Figure 1:**
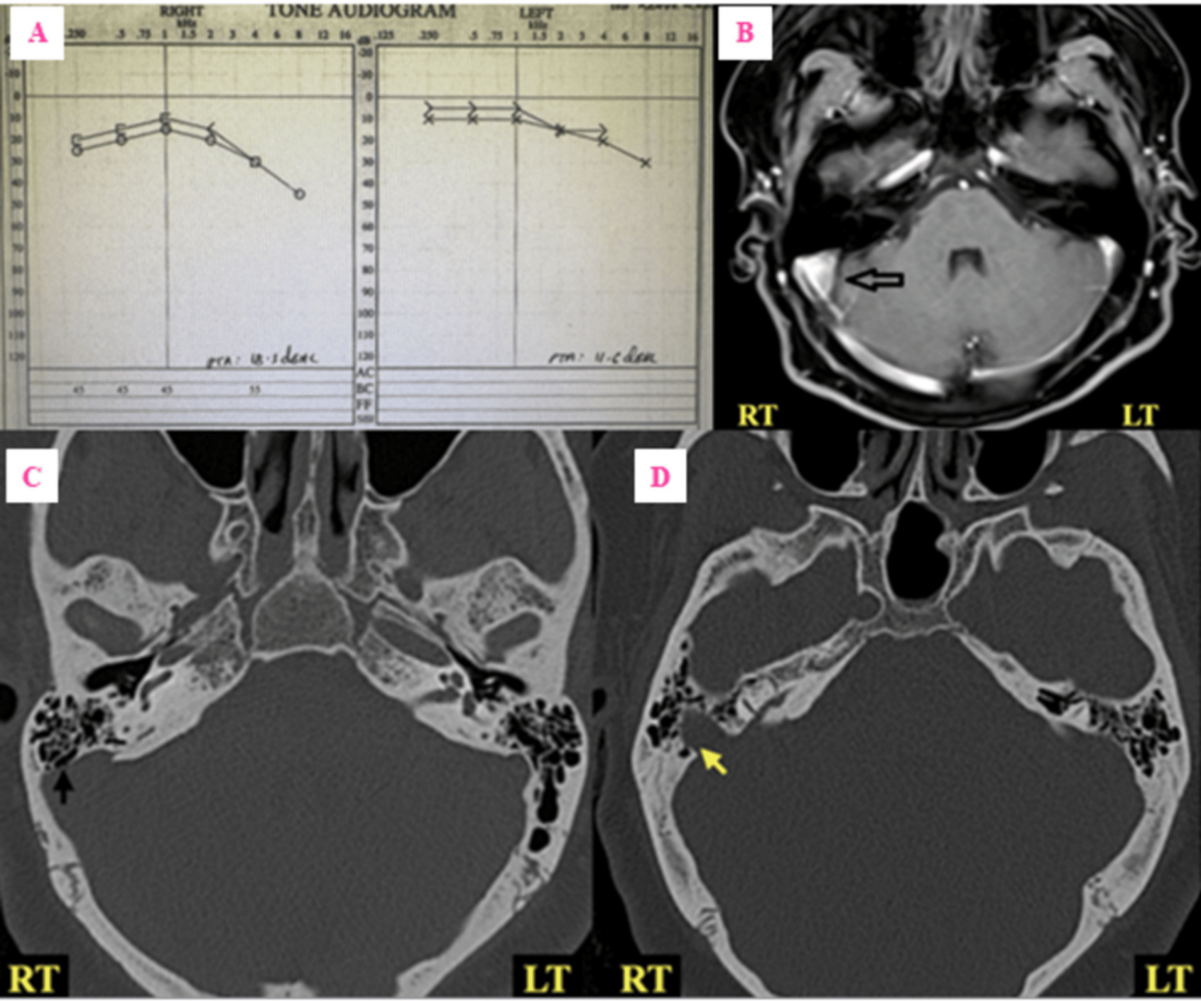
(A) Pure tone audiogram of right and left ears, (B) MRI, and (C, D) CT displaying right-sided unilateral sigmoid sinus diverticulum and sinus plate dehiscence (A) Pure-tone audiogram showing moderate sensorineural hearing loss involving both low and high frequencies on the right side and minimal high-frequency sensorineural hearing loss on the left side. (B) Contrast-enhanced T1 fat-suppressed image in axial plane displaying right-sided sigmoid sinus diverticulum (arrow) with focal protrusion of sigmoid sinus towards the mastoid air cells. (C) Absence of normal bony coverage (arrow) of the right sigmoid sinus with air-on-sinus sign. There is a normal bony covering over the left sigmoid sinus. (D) Axial high-resolution CT (HRCT) image depicting the right-sided sigmoid sinus diverticulum (arrow).

Physical examination of the head and neck was unremarkable. The external examination of both auricles appeared normal. Otoscopic evaluation revealed a normal external auditory canal and intact tympanic membranes bilaterally. Neurological and vestibular examinations were within normal limits. Audiometric evaluation demonstrated sensorineural hearing loss involving both high and low frequencies in the right ear, with a plateau-type configuration (Figure 1A). There was no conductive hearing loss. Tympanometry showed normal middle ear pressure and compliance. No other systemic abnormalities were observed on general examination.

**Figure 2:**
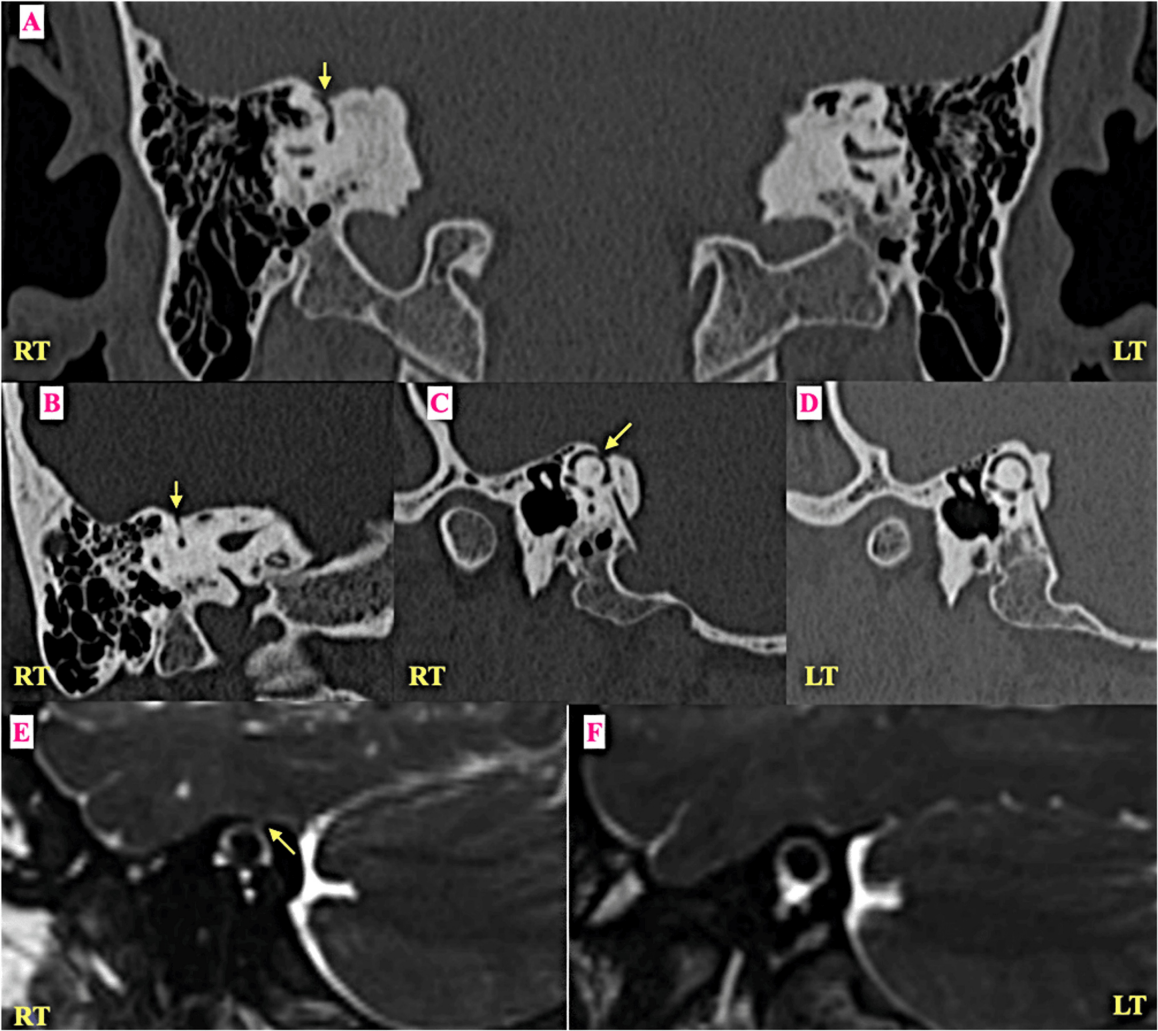
Right-side unilateral dehiscence of superior semicircular canal (A) Coronal high-resolution CT (HRCT) image of the temporal bone displaying a small area of bony dehiscence (yellow arrow) over the superior semicircular canal on the right side. A normal thick bony rim is seen overlying the left superior semicircular canal. (B) HRCT image of the right temporal bone in “Stenver’s plane” showing the bony dehiscence (yellow arrow) over the right superior semicircular canal. (C) HRCT image of the right temporal bone in “Poschl’s plane” showing a small area of bony dehiscence (yellow arrow) over the right superior semicircular canal. (D) The HRCT image of the left temporal bone in “Poschl’s plane” showing a normal thick bony cover over the left superior semicircular canal. (E) 2D reformation of a 3D Constructive Interference in Steady State (CISS) image of the right temporal bone in “Poschl’s plane” showing a small area of bony dehiscence (yellow arrow) over the right superior semicircular canal. (F) 2D reformation of a 3D CISS image of the left temporal bone in “Poschl’s plane” showing a normal bony covering over the left superior semicircular canal.

High-resolution computed tomography (HRCT) of the temporal bones revealed dehiscence of the right sigmoid sinus plate with an associated diverticulum (Figure 1C). Additionally, a focal bony defect was identified in the right superior semicircular canal (Figure 2), which was better appreciated on reformatted images in Poschl’s (parallel) and Stenver’s (perpendicular) planes. Contrast-enhanced MRI of the brain and internal auditory canals confirmed the presence of a right-sided sigmoid sinus diverticulum adjacent to the area of bony dehiscence (Figure 1B). No vascular malformations or intracranial masses were identified. A diagnosis of coexistent SSCD and SSPD contributing to pulsatile tinnitus was established.

In light of the absence of disabling vestibular symptoms, conservative management was recommended. This included advising the patient to avoid Valsalva manoeuvres and high-impact activities that could exacerbate symptoms. At the three-month follow-up, the patient reported stable symptoms with no significant worsening. Surgical options, including canal plugging for SSCD and sigmoid sinus plate resurfacing or reconstruction for SSPD, were discussed as potential future interventions should symptoms become debilitating or significantly impair her quality of life.

## Discussion

Pulsatile tinnitus can arise from a range of etiologies, including vascular anomalies, venous sinus abnormalities, and temporal bone defects such as superior semicircular canal dehiscence (SSCD) and sigmoid sinus plate dehiscence (SSPD). Unlike non-pulsatile tinnitus, pulsatile tinnitus is typically synchronous with the patient’s heartbeat, often raising suspicion for underlying vascular or anatomical abnormalities. This case highlights the complex interplay between structural and vascular anomalies within the temporal bone and their auditory consequences.

Superior semicircular canal dehiscence (SSCD) can present with either vestibular or auditory symptoms. It is commonly linked to low-frequency conductive hearing loss, caused by a 'third-window' effect that disrupts normal sound transmission in the cochlea [4]. However, this hearing loss is not always present. According to various published studies [5,6], audiometric findings in SSCD can range from normal hearing to conductive or sensorineural hearing loss. In contrast, high-frequency sensorineural hearing loss may result from vascular noise transmission caused by sigmoid sinus plate dehiscence, where turbulent blood flow interferes with cochlear hair cell function [1]. The coexistence of both conditions in a single patient is rare but emphasizes the importance of thorough imaging and audiological evaluation.

In patients presenting with both pulsatile tinnitus and hearing loss, high-resolution computed tomography (CT) of the temporal bone is considered the initial imaging modality of choice to assess for osseous abnormalities [7]. The most common bony defect implicated in pulsatile tinnitus is dehiscence of the sigmoid sinus wall. This wall is composed of thin cortical bone separating the sigmoid sinus from the adjacent mastoid air cells. When dehiscent, it produces the characteristic “air-on-sinus” sign and may be associated with sigmoid sinus diverticula, which are better evaluated with CT or MR venography.

SSCD is another important osseous cause of pulsatile tinnitus, characterized by a defect in the bony covering over the superior aspect of the superior semicircular canal. The dehiscence is best visualized on reformatted CT images in planes parallel (Poschl’s) and perpendicular (Stenver’s) to the canal [8,9]. MRI sequences using fast imaging techniques such as Fast Imaging Employing Steady-state Acquisition (FIESTA) or Constructive Interference in Steady State (CISS) have demonstrated 100% sensitivity and negative predictive value, with 96.5% specificity and 61% positive predictive value compared with CT in the evaluation of SSCD, suggesting that MRI may be sufficient to exclude the diagnosis in some cases [10].

Management should be guided by symptom severity, with conservative measures often appropriate for mild cases. Multidisciplinary care involving otologists, radiologists, and vascular specialists is essential for comprehensive evaluation and management.

## Conclusions

This rare case of concurrent superior semicircular canal dehiscence (SSCD) and sigmoid sinus plate dehiscence (SSPD) in a patient with pulsatile tinnitus underscores the importance of thorough clinical and comprehensive radiological evaluation. Given that pulsatile tinnitus can result from a wide spectrum of pathological conditions, a systematic workup is essential to identify the precise etiology. Accurate diagnosis enables the formulation of a tailored treatment plan, which is critical for effective symptom management and improved patient outcomes.
